# Temporal Changes in Faecal Microbiota Composition and Diversity in Dairy Cows Supplemented with a *Lactobacillus*-Based Direct-Fed Microbial

**DOI:** 10.3390/ani14233437

**Published:** 2024-11-27

**Authors:** Bronwyn E. Campbell, Mohammad Mahmudul Hassan, Robert J. Moore, Timothy Olchowy, Shahab Ranjbar, Martin Soust, Orlando Ramirez-Garzon, Rafat Al Jassim, John I. Alawneh

**Affiliations:** 1 School of Science, RMIT University, Bundoora, Melbourne, VIC 3068, Australia; bronwyn.campbell@rmit.edu.au (B.E.C.); rob.moore@rmit.edu.au (R.J.M.); 2 School of Veterinary Science, The University of Queensland, Gatton, QLD 4343, Australia; s.ranjbar@uq.edu.au; 3 Faculty of Veterinary Medicine, University of Calgary, Calgary, AB T3R 1J3, Canada; timothy.olchowy@ucalgary.ca; 4 Terragen Biotech Pty Ltd., Coolum Beach, QLD 4573, Australia; msoust@me.com (M.S.); o.ramirez@uq.edu.au (O.R.-G.); 5 Queensland Alliance for Agriculture and Food Innovation, Brisbane, QLD 4072, Australia; r.aljassim@uq.edu.au

**Keywords:** faecal microbiota, dairy cows, DFM, microbial diversity, temporal changes

## Abstract

The rumen microbiota of dairy cows plays a crucial role in fermenting fibrous material, essential for nutrient extraction and overall productivity, detoxification of anti-nutritional toxic compounds, synthesis of vital nutrients, and is essential for optimal animal health. This study investigated the impact of *Lentilactobacillus*-, *Lactocaseibacillus*-, and *Lacticaseibacillus*-based direct-fed microbial (DFM) supplementation on dairy cows’ faecal microbial composition and diversity. The study was carried out on a commercial dairy farm using 50 Holstein-Friesian cows randomly assigned into control (CON) and treatment (TRT) groups. Faecal samples were collected directly from the rectum every two months from September 2021 to January 2023. The bacterial 16S rRNA gene and fungal ITS-1 regions were amplified, sequenced, and analysed. Microbial diversity was assessed through alpha- and beta-diversity metrics. Linear discriminant analysis effect size (LEfSe) was performed to identify which taxa were driving the changes seen in the microbiota over time and treatment. *Bacteroidaceae* were the most prevalent bacterial family, followed by *Lachnospiraceae* and *Muribaculaceae* in both CON and TRT cows. *Ascomycota*, *Basidiomycota*, and *Mucoromycota* were the dominant three fungal phyla in the faeces of both CON and TRT cows. Bacterial genera *Fructilactobacillus* was abundant in the CON and *Absicoccus* in the TRT groups. Fungal taxa *Chaetothryriales*_incertae_sedis and *Pseudomentella* were absent in the faeces of TRT cows. Significant temporal and specific taxonomic differences were observed between the CON and TRT groups. The study’s findings underscore the dynamic nature of microbial communities and the importance of targeted dietary interventions. Further research is necessary to elucidate these microbial shifts, long-term impacts, and functional implications, aiming to optimise ruminant nutrition and enhance dairy cow performance.

## Simple Summary

This study investigated the effects of a *Lactobacillus*-based direct-fed microbial (DFM) supplement on the faecal microbiota of dairy cows over 16 months. Significant temporal changes in microbial diversity and composition between experimental groups were observed. DFM supplementation can potentially improve dairy cow health and productivity by enhancing fermentation efficiency and nutrient absorption. Understanding these microbial shifts contributes to sustainable agricultural practices by optimising dietary interventions, promoting animal welfare, and reducing environmental impact through improved feed utilisation. This research highlights the potential for microbiota management to advance dairy industry sustainability and efficiency.

## 1. Introduction

The rumen of dairy cows is essential for fermenting and digesting plant material, and to enable nutrient extraction vital for the animal’s growth and productivity. The ruminal microbiota, a complex mix of bacteria, archaea, and eukaryotes, drives this process [1]. In addition to its primary role of fermenting fibrous constituents of the diet, these microorganisms synthesise essential nutrients not found in the animal’s diet [2]. They also destroy anti-nutritional factors [3]. About 60% of the rumen microbiota is bacteria, primarily from the phyla Firmicutes and Bacteroidetes, each making up around 30% of the community [4,5]. Archaea account for about 0.3 to 3.3% of the microbiota [6], while eukaryotes, mainly protists, comprise 30–40% of rumen biomass [7], with fungi making up around 1%. The rumen and faecal microbiota exist in association with the solid and liquid phases of digesta, with over 80% residing in the solid phase [4,8]. Dominant bacterial genera include *Succiniclasticum*, *Lachnospira*, and *Ruminococcus* from the phylum Firmicutes, and *Prevotella* from the phylum Bacteroidetes [9,10]. These bacteria play key roles in the breakdown and fermentation of plant material within the rumen. The faecal microbiota offers important insights into the overall gut health of cows but is less frequently studied than ruminal contents. The DFMs can stimulate mechanisms such as enhanced fibre digestion through cellulolytic enzyme production, increased volatile fatty acid synthesis, improved nitrogen utilisation, and the modulation of host immune responses, ultimately promoting better gut health and nutrient absorption [2,11]. It can differ significantly from the ruminal microbiota, highlighting different stages of digestion and the influence of external factors [9]. These differences provide a broader understanding of the cow’s digestive health and gastrointestinal tract microbial dynamics.

Recent studies emphasise the crucial role of microbiota in livestock health and productivity [1,4,5]. Interactions between diet and microbiota significantly affect the composition of the gut microbiota [11,12]. Dietary interventions have been shown to modulate microbial communities, promoting improved health outcomes in livestock [13]. Direct-fed microbial (DFM) supplements, such as *Bifidobacterium*, *Enterococcus*, *Streptococcus*, *Prevotella*, *Bacillus*, and *Lactobacillus*, are introduced to modulate the gut microbiota and aim to boost the health and productivity of dairy cows [14,15]. In beef cattle, significant observable changes in faecal microbiota profiles have been noted, in terms of relative abundances and alpha-diversity metrics, to occur two days post feedlot induction. Beta-diversity analysis demonstrated a significant decrease in the phylogenetic similarity between samples [16]. These trends suggest the presence of a short-term reduction in microbial diversity and decreased similarity between animals, which could potentially impact gut health and disease resistance [17,18]. Such shifts in microbial populations can influence digestion efficiency, thus highlighting the importance of careful management of dietary transitions [19].

Previous studies have demonstrated enhancements in animal productivity when the DFM formulation utilised in this trial was administered either as a topdressing on pasture [20] or incorporated into the partial mixed rations of the animals [21]. Therefore, it is hypothesised in this study that the DFM influences microbial populations such as bacteria, archaea, fungi, and eukaryotes in the digestive tract, potentially enhancing fermentation efficiency and nutrient absorption. This study aimed to comprehensively analyse the bacterial and fungal communities in faecal samples of DFM-supplemented and control cows. By comparing these microbial communities over time, the research seeks to understand the temporal dynamics and identify specific taxa affected by the DFM supplementation. The outcome provides insights into how supplementing DFM could influence microbial composition and diversity in dairy cows.

## 2. Materials and Methods

### 2.1. Study Location, Study Herd, and Study Animals

Full details of the study location, herd, and design have been published elsewhere [21]. This study was conducted at Harrisville, Queensland, Australia from September 2021 to January 2023 on a commercial dairy farm. Fifty cows, twenty-five in the treated (TRT) group and twenty-five in the control (CON) group, were randomly selected from approximately 350 Holstein cows in a milking herd. The sample size of the study animals was determined by assuming a difference of 50% in microbiome structure, an alpha of 2.5%, a power of 80%, no change in faecal microbiome structure in the CON group, a change in the faecal microbiome structure of 50% in the TRT group, and a difference of 15% is negligible. Study animals were selected based on parity and days in milk (DIM). Each group was maintained separately, including the feeding and milking period. Cows were fed a mixed ration during the day and allowed to graze the pasture at night. Partial mixed rations (PMRs) included maize, soybean and barley silage, lucerne hay, canola meal, and barley or wheat grain. Barley and wheat grain (1.5 kg as fed) was fed to the cows twice a day, and the pasture consisted of ryegrass or kikuyu (up to 6 kgDM). Water was provided ad libitum. The cows in the DFM group were provided the DFM as a top dressing of their mixed ration at a rate of 10 mL/cow/day. The DFM (Mylo^®^, Terragen Biotech, Coast, QLD, Australia) contained three strains of live bacteria: *Lentilactobacillus buchneri* Lb23, *Lactocaseibacillus casei* Lz26, and *Lacticaseibacillus paracasei* T9, each at approximately 3.5 × 109 CFU/mL.

### 2.2. Sample Collection

Faecal samples were collected from all cows approximately every two months from September 2021 to January 2023, totalling eight time points. The sampling intensity was informed by convenience and the anticipated changes in herd nutrition and structure. The samples were taken directly from the rectum using a protective glove at approximately 9:00 a.m., three hours after feeding. A subsample of the collected faeces (3–4 g) was placed into sterile 5 mL polypropylene flat bottom tubes (Interpath, Melbourne, Australia) for faecal microbiota analysis.

### 2.3. DNA Extraction and PCR

Samples of 100 mg of faeces were weighed and processed for total DNA extraction using the Maxwell^®^ RSC Faecal Microbiome DNA Kit (Promega, Fitchburg, WI, USA) according to the manufacturer’s protocol (Maxwell^®^ RSC Fecal Microbiome DNA Kit Technical Manual, Promega). A bead-beating step was added before processing via the Maxwell kit; tubes were bead-beaten using a FastPrep 24 instrument (MP Biomedicals, Irvine, CA, USA) for one minute at 4 m/s, followed by a five-minute break, and then beaten for another minute. The extracted DNA was subjected to PCR to amplify the bacterial V3–V4 region of 16S rRNA genes and the fungal internal transcribed spacer 1 (ITS-1) regions [22,23]. Forward and reverse sequencing indexes were added to the amplicons using 96 forward indexes from the Nextera XT Index 1 plate (Illumina, New York, NY, USA) and three reverse indexes, creating 288 unique index combinations. Negative controls were included in both the target amplification PCR and in the PCR for indexing.

### 2.4. Amplicon Purification, Library Mixing, and Sequencing

This protocol followed the Illumina 16S Metagenomic Sequencing Library preparation document (#15044223). Paired-end sequencing (2 × 300 bp) was performed using the MiSeq Reagent Kit v3 (600 cycle, Illumina, New York, NY, USA).

### 2.5. Quality Control and Sequence Read Counts

Read counts were performed using the Linux command line “echo $(zcat sequence_file.fastq.gz|wc -l)/4|bc”. A minimum read count of 30,000 for all bacteria and 4000 for fungi was targeted to provide a sufficient representation of the taxa present in the samples. Those samples that fell below the required sequencing depth (*n* = 71) were re-sequenced, starting from the original template. The quality of the reads and assessment of the need for trimming was determined using FastQC, Version 0.12.0 (http://www.bioinformatics.babraham.ac.uk/projects/fastqc/, accessed on 24 October 2024).

### 2.6. Statistical Analysis

Sequence files were denoised and trimmed using the DADA2 plugin [24] within the QIIME2 platform [25]. Amplicon sequence variants (ASV) were filtered by sample and feature and then summarised. Final representative sequences were compared against the 16S GreenGenes2 [26] and ITS-1 UNITE [27,28] databases using the feature-classifier. Phylogenetic analysis used multiple sequence alignment and masking to group sequences and remove ambiguous sequences. FastTree, Version 2.1 was then used to construct the phylogeny from filtered sequences. Four files were used for further analysis in MicrobiomeAnalyst Version 2.0 (https://www.microbiomeanalyst.ca/, accessed on 24 October 2024), including the feature table, taxonomy, metadata as comma-delimited (.csv), and the phylogenetic tree in newick format (.nwk).

Alpha and beta-diversity metrics were calculated to assess the microbial diversity within and between samples. Alpha-diversity analysis was performed to statistically test diversity differences within samples (overall and within each time point). This allowed the determination of the species richness and evenness within a sample type and experimental days. The methodologies used in the analysis measured the Chao1 [29], Observed [30], and Shannon [31] indexes, and a *t*-test was conducted to estimate the level of significance. Probability values at an alpha ≤ 0.05 were considered statistically significant. Samples were compared by treatment and experimental time point (month). The analysis was performed at the genus level. Beta-diversity analysis via principal component analysis (PCoA; [32]) and non-metric multidimensional scaling (NMDS; [33]) was performed to compare treatment and experimental time points (months). This allowed testing of whether differences between samples were statistically significant.

The linear discriminant analysis (LDA) effect size (LEfSe) method was used to identify which taxa were most responsible for the differences between groups. This analysis determined the differentially abundant taxa between TRT and CON cows. Statistical significance was assessed, with an alpha ≤ 0.05 as significant. The analysis results reported were limited to the genus level. All analyses were performed using MicrobiomeAnalyst Version 2.0 (https://www.microbiomeanalyst.ca/, accessed on 24 October 2024). Alpha-diversity and core microbiota data were plotted using GraphPad Prism Version 10 (Dotmatics), whilst beta-diversity plots were produced using the ggplot2 package (v3.5.1, [34]) implemented within Rstudio (Build 421, 2023.06.0 [35]).

## 3. Results

### 3.1. Bacterial Microbiota

**Figure 1:**
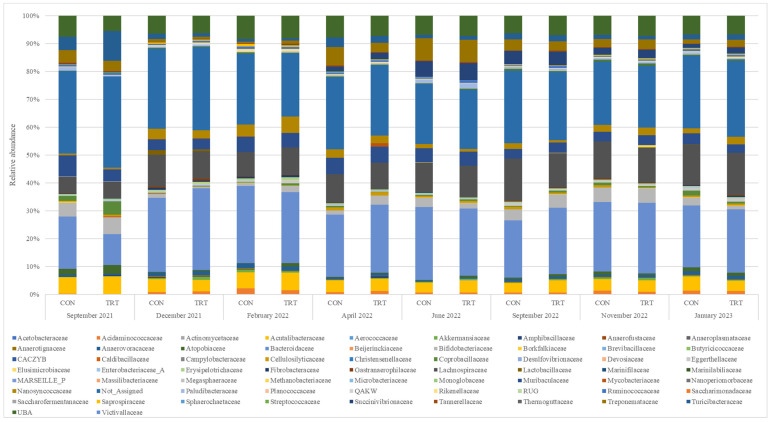
Relative abundance of bacterial families in faeces for control (CON) and DFM-treated (TRT) groups throughout the study period.

The sequencing of bacterial faecal sample DNA produced a total of 21,320,314 reads, with reads per sample ranging from 30,854 to 110,493 (mean 56,854). For fungal analysis, sequencing generated 11,575,937 reads, with reads ranging from 4115 to 98,762 per sample (mean 26,983). The most abundant families in both CON and TRT cows differed by only 1%. The dominant bacterial family was *Bacteroidaceae* (44% in CON and 43% in TRT cows), followed by *Lachnospiraceae* (21% and 20%), *Muribaculaceae* (9% and 8%), and *Turicibacteraceae* (4% and 5%). Both groups had equal abundances of *Bifidobacteriaceae* (6%), *Ruminococcaceae* (1%), *Akkermansiaceae* (1%), *Rikenellaceae* (1%), *Erysipelotrichaceae* (1%), and *Christensenellaceae* (0.002%). Additionally, low abundances of *Methanobacteriaceae* (0.2%) were detected in TRT cows compared to CON cows. The relative abundances of various bacterial families were analysed across different months from September 2021 to January 2023, providing insights into the temporal dynamics of the microbial communities. The proportions of dominant families such as *Acetobacteraceae*, *Actinomycetaceae*, and *Bifidobacteriaceae* showed notable fluctuations over time (Figure 1). We analysed 31 genera within the *Lactobacillaceae* family over time and across treatments in the faecal samples. The results showed a consistently low abundance of lactobacilli throughout the study period, which is consistent with our analysis. As with other microbiota analysis studies, we applied a cutoff threshold of less than 10% for microbiota composition, ensuring the focus remained on crucial contributors within the microbiome.

**Figure 2:**
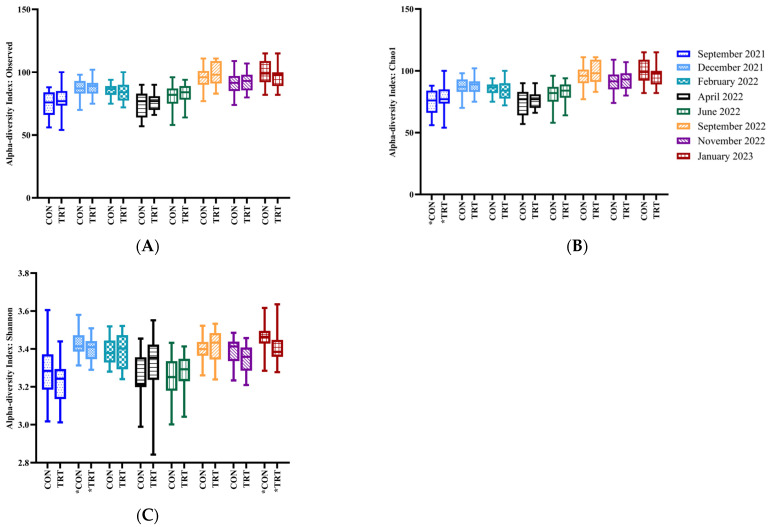
Bacterial alpha diversity analysis (genus level) over time in faeces from Control (CON) and DFM-treated (TRT) experimental groups. Observed (**A**) and Chao1 (**B**) for Shannon index (**C**). Pooled data for overall differences between experimental groups disregarding the effect of the calendar month: Observed and Chao1, *p*-value: <0.001, *t*-test: 46.5; Shannon, *p*-value: <0.001, *t*-test: −25.2. Asterisk (*) on *x*-axis below experimental group denotes *p* < 0.05 between experimental groups for a given calendar month.

**Figure 3:**
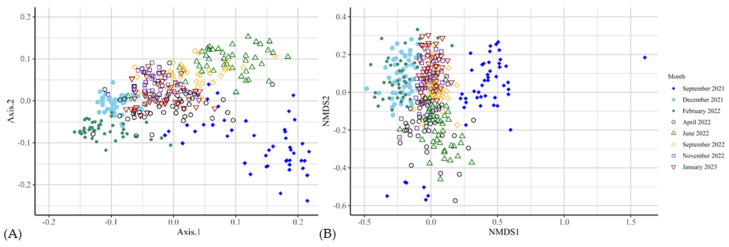
Bacterial beta-diversity (genus level) analysis via (**A**) principal component analysis and (**B**) non-metric multidimensional scaling of faeces across eight time points from September 2021 to January 2023. Microbial diversity differed significantly over the 16 months of the trial.

Alpha-diversity differed within experimental groups for September 2021 (Observed, *p* = 0.002; Chao1, *p* = 0.002; Figure 2). The Shannon index differed between the CON and TRT groups in December 2021 (*p* = 0.02) and January 2023 (*p* = 0.002). Other months did not exhibit changes (*p* > 0.05) across the indices (Figure 2; Supplementary Table S1). Faecal microbial diversity tested using beta-diversity analysis differed significantly at the genus level over the 16 months of the trial (Figure 3; Supplementary Figure S1; Supplementary Table S2).

**Figure 4:**
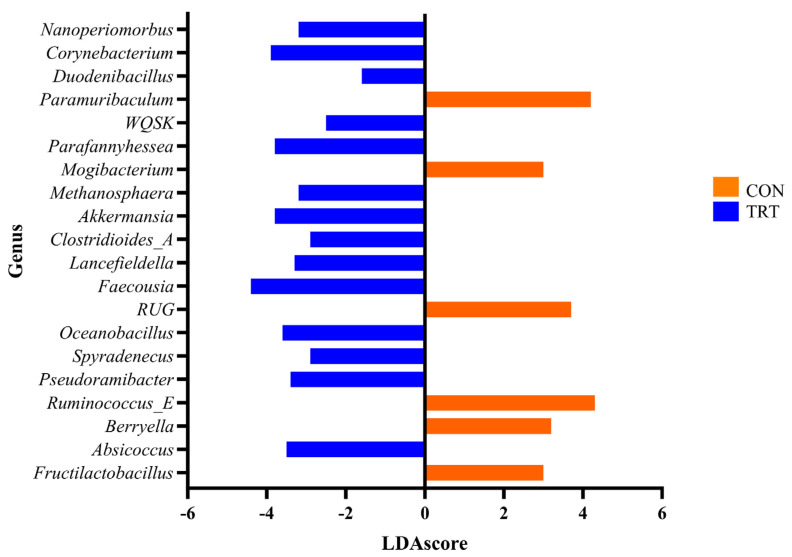
Linear discriminant analysis effect size (LEfSe) analysis of the top twenty genera from faeces from Control (CON) compared with DFM-supplemented (TRT) experimental group. LDAscore refers to the linear discriminant analysis scores.

Twenty genera were found by LEfSe analysis to differ (*p* < 0.05) in faeces from CON compared to TRT cows. *Fructilactobacillus* (*p* < 0.001; LDAscore = 3.0) and *Berryella* (*p* < 0.001; LDAscore = 3.2) were more abundant in the CON group, while *Absicoccus* (*p* < 0.001; LDAscore = −3.5), *Ruminococcus*_E (*p* < 0.001; LDAscore = 4.3), and *Pseudoramibacter* (*p* < 0.001; LDAscore = −3.4) were more abundant in the TRT group (Figure 4).

*Paraprevotella*, *Turicibacter*, and *Phocaeicola*_A_858004 were the top genera with the most significant differences over time between the CON and TRT groups. *Paraprevotella* had a higher relative abundance in CON cows in September 2021 (*p* < 0.001), while *Turicibacter* showed a higher abundance in TRT cows (*p* < 0.001). *Phocaeicola*_A_858004 consistently appeared in the analysis, showing significant differences in December 2021, September 2022, and February 2022, with its highest abundance in TRT cows in December 2021 (Supplementary Table S3).

### 3.2. Fungal Microbiota

**Figure 5:**
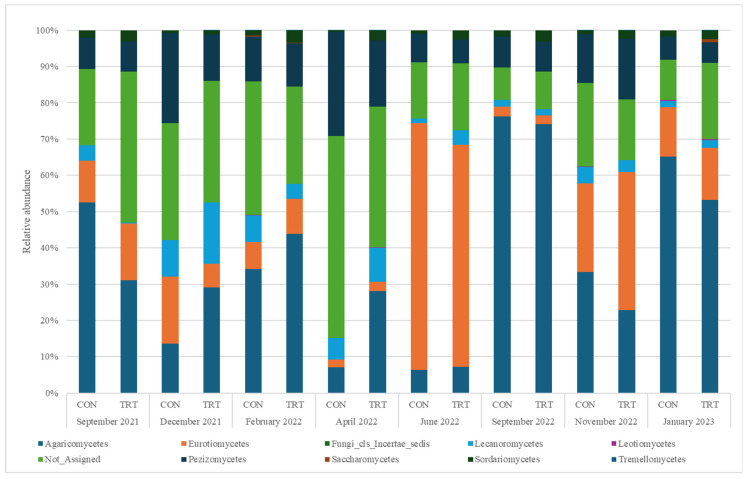
Relative abundance of fungal classes in faeces for control (CON) and treatment (TRT) groups throughout the study period.

The most abundant fungal taxa observed were *Ascomycota*, *Basidiomycota*, and *Mucoromycota*. *Ascomycota* had the highest relative abundance in CON and TRT groups, with noticeable variations between CON and TRT groups over time. *Basidiomycota* and *Mucoromycota* also displayed significant differences in their relative abundance between the groups, reflecting the impact of the treatment on the fungal composition in the faeces (Figure 5).

**Figure 6:**
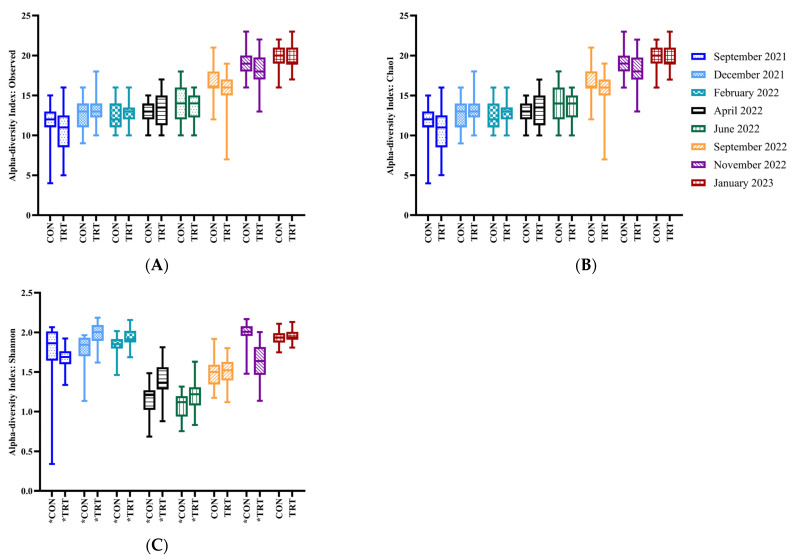
Alpha-diversity analysis (genus-level) of the fungal composition of faeces over time from Control (CON) compared with DFM-treated (TRT) cows. Observed (**A**) and Chao1 (**B**) for Shannon index (**C**). Pooled data for overall differences between experimental groups disregarding the effect of the calendar month: Observed and Chao1, *p*-value 0.32, *t*-test: 0.99; Shannon, *p*-value: 0.23, *t*-test: −1.2. Asterisk (*) on *x*-axis below experimental group denotes *p* < 0.05 between experimental groups for a given calendar month.

**Figure 7:**
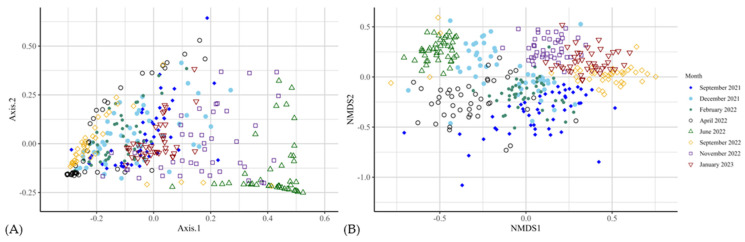
Bacterial beta-diversity (genus level) analysis of fungi of faeces across eight time points from September 2021 to January 2023 (**A**,**B**), using Microbial diversity differed significantly over the 16 months of the trial.

The fungal alpha-diversity at the genus level differed between CON and TRT groups (Shannon diversity index *p* ≤ 0.05 in six out of eight months). Observed (*p* > 0.05) and Chao1 (*p* > 0.05) indices did not differ between experimental groups (Figure 6; Supplementary Table S4). Beta-diversity was significantly different between CON and TRT groups across most time points, except for April 2022 (*p* = 0.14; Figure 7 and Figure S2; Supplementary Table S5).

**Figure 8:**
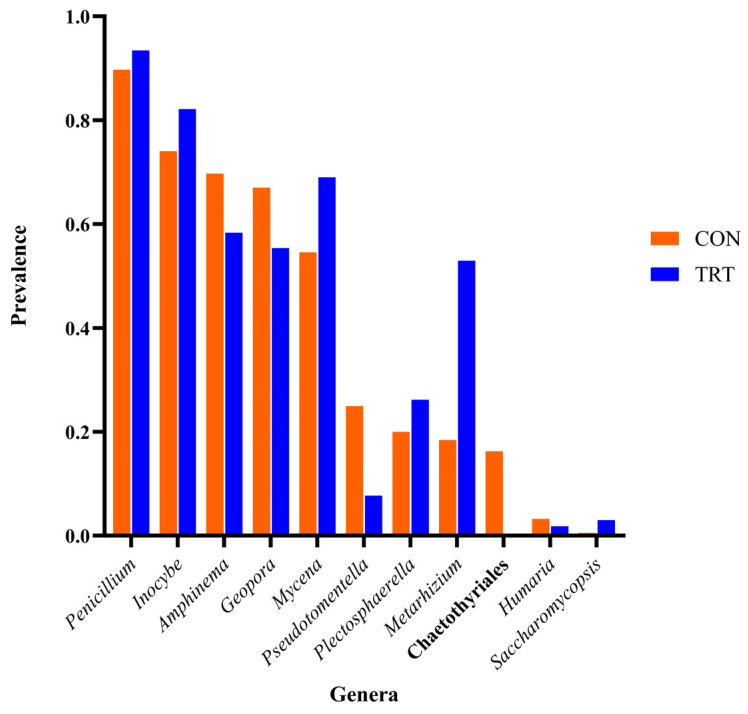
Prevalences of fungal genera found as the core microbiota of the faeces of Control (CON) and DFM-supplemented (TRT) cows.

The fungal core microbiota of both TRT and CON cows, including the prevalence of each genus within them, are shown in Figure 8. The main difference between the core fungal microbiota of CON and TRT is the absence of two taxa, *Chaetothryriales* and *Pseudomentella*, in the TRT cows.

## 4. Discussion

The findings of this study offer valuable insights into how a *Lentilactobacillus*-, *Lactocaseibacillus*-, and *Lacticaseibacillus*-based supplementation affects faecal microbiota composition and diversity in dairy cows, with potential implications for animal productivity. Prior studies on cows administering a DFM supplement demonstrated improved live weight and increased milk production [21,36,37]. The results of the current study emphasise the complexity of faecal microbiota and feed additives’ potential to modulate those communities. Additionally, the study demonstrates the dynamic nature of microbiota in the faeces of dairy cows and the significant impact of DFM supplementation over time. It is hypothesised that some of the observed changes in microbiota composition, specifically bacterial and fungal microbiota, between the CON and TRT cows may have a role in improving the performance of the TRT cows.

The dominance of *Firmicutes* and *Bacteroidetes* in the faecal microbiota aligns with previous research indicating the stability of these core microbial groups [38,39,40]. The observed temporal variations highlight the impact of external factors such as diet, environment, and management practices on microbial communities. The lack of significant differences in alpha-diversity within treated and control cows showed that the DFM treatment did not drastically change overall bacterial and fungal microbial diversity. This indicates that the supplement’s impact may be more nuanced, affecting a limited number of very specific taxa rather than the entire microbial community [41,42]. The beta-diversity analysis and LEfSe results support the hypothesis that DFM treatment just affected specific microbial taxa. This is consistent with other studies that showed that while overall diversity remained stable, certain bacteria were influenced by the DFM supplement [23,43]. Such types of changes in microbial composition could influence fermentation efficiency and nutrient absorption in cows. This could improve animal health by optimising their digestive processes. Notwithstanding, our study used faecal samples as a proxy for rumen microbial dynamics. Since faecal microbiota primarily reflects the hindgut community, the observed changes may not accurately represent the rumen. Direct rumen sampling is needed to fully understand the effects of DFM on the digestive microbiota.

The presence of significant differences in bacterial beta-diversity between DFM-supplemented and control cows suggests that DFM supplementation had a significant impact on faecal microbiota. This finding aligns with other studies on the effects of feed additives on ruminal microbiota, which often show subtle rather than dramatic changes in microbial diversity [44]. The results show that the DFM supplement influenced the relative abundance of specific faecal microbial taxa. It is notable that certain genera, such as *Bacteroidaceae*, known for their involvement in protein digestion and starch utilisation, exhibited significant changes in abundance. This observation aligns with prior research indicating that feed additives can selectively promote or inhibit specific microbial populations, altering the overall fermentation process [45]. Recent studies have corroborated similar effects with other dietary interventions and feed additives, emphasising the dynamic nature of microbial responses to external interventions. The modulation of microbial communities holds considerable implications for animal health. A potential enhancement of nutrient absorption and immune responses highlights the importance of understanding and optimising microbial balance in ruminant nutritional management [46]. Alterations in the abundance of other key genera, such as *Ruminococcus* and *Pseudoramibacter*, impacted the production of short-chain fatty acids, which are crucial for the energy metabolism of dairy cows [47]. These findings highlight the intricate interplay between microbial composition and metabolic processes in the rumen, underscoring the importance of understanding and optimising microbial communities for enhanced animal health and productivity [6,47].

The fungal microbiota analysis uncovered a notable proportion of unclassified ASVs, emphasising the necessity for more comprehensive fungal databases and enhanced taxonomic classification methods [48]. The significant temporal variations observed in fungal diversity underscore the importance of fungi in faecal microbiota dynamics, implying a potential influence on dairy cows’ health [2,49]. Although DFM had no significant effect on fungal diversity, the core fungal microbiota of CON cows differed from that of TRT cows primarily in the absence of two taxa: *Chaetothryriales*_incertae_sedis and *Pseudomentella*. Generally, species within the order *Chaetothryriales* are predominantly epiphytic or saprobic, residing on decaying organic matter [50]. In contrast, *Pseudomentella* species were commonly found in soil and are associated with mycorrhizal communities of plants [51]. These differences suggest potential shifts in environmental interactions and nutrient cycling within the gastrointestinal microbiota of dairy cows receiving the DFM treatment.

The temporal changes observed were essential for comprehending the microbial community’s response to oral DFM supplementation. The study revealed significant temporal variations in microbial diversity, aligning with previous research highlighting the dynamic nature of the faecal microbiota [16,52]. However, these changes suggest that the relative abundances of core faecal microbial groups can vary over time, and if a similar response occurs in the rumen, the impact on the cows’ digestion will be an essential outcome [53,54]. Changes in faecal microbiota may not accurately represent what is happening in the rumen, but this underscores the importance of ongoing monitoring and management practices to optimise microbial balance and support bovine health and productivity. *Lactobacillus*-based DFMs promote a stable rumen environment by reducing harmful pathogens, optimising pH, and increasing volatile fatty acid (VFA) production, thereby improving digestion and immune function [55]. The biological significance of microbial changes in cow health and productivity is evident through enhanced nutrient absorption, reduced disease incidence, and improved feed efficiency, directly impacting milk yield and growth performance [11]. These effects are attributed to the ability of *Lactobacillus* species to modulate gut microbiota, outcompete pathogens, and produce antimicrobial peptides [14].

To advance the findings of this study, future research should prioritise longitudinal studies to investigate the enduring effects of the DFM supplement on microbial community structure and function of the rumen and rectum over extended periods. Functional analysis, employing metagenomic and metabolomic approaches, would offer deeper insights into the functional roles of affected microbial taxa. Given its significance in ruminal ecology, enhancing fungal databases and taxonomic classification methods is crucial for accurately characterising the rumen and rectal fungal microbiota. Such efforts would collectively contribute to a more thorough understanding of how DFM supplementation shapes gastrointestinal tract microbial communities and their functional dynamics in dairy cows. Ultimately, this knowledge can inform targeted interventions to optimise animal health, productivity, and welfare in ruminant production systems. Further research is needed to examine the faecal microbiota and the connection of rumen microbiota studies with animal performance. It is also crucial to determine the similarity between faecal and rumen microbiota and whether faecal microbiota accurately represents the ruminal microbiota. Expanding the scope of this research could offer valuable insights into the factors affecting gastrointestinal tract microbial dynamics in dairy cows, helping to develop targeted management practices for optimising animal health and productivity.

## 5. Conclusions

This study highlights the impact of direct-fed microbial supplementation on dairy cows’ faecal microbiota composition and diversity. While overall alpha-diversity remained stable, significant temporal and specific taxonomic changes were observed, particularly among bacterial families such as *Bacteroidaceae*, which are known for digestion and absorption. These findings suggest that DFMs can modulate specific microbial populations, potentially enhancing fermentation efficiency and nutrient absorption. Significant temporal variations in fungal diversity also emphasise the need for better fungal databases and classification methods. Further research is necessary to understand the long-term effects and functional implications of these microbial shifts before optimised dietary interventions to improve the health and productivity of dairy cows can become a routine farm-specific health management tool. Despite its potential advantages, the specific effects of this DFM on the microbiota composition and diversity in dairy cows remain largely unexplored. Further research is needed to understand its full impact and efficacy.

## Data Availability

If necessary, a corresponding author will provide data on request.

## Supplementary Materials

The following supporting information can be downloaded at: https://www.mdpi.com/article/10.3390/ani14233437/s1, Figure S1: Relative abundance of bacterial families in faeces for CON and TRT groups throughout the study period; Figure S2: Bacterial beta-diversity (genus level) analysis of faeces from TRT and CON cows; Figure S3: Relative abundance of fungal classes in faeces for CON and treatment TRT groups throughout the study period; Figure S4: Fungal Beta-diversity analysis (genus-level) of fungal diversity between CON (orange) and TRT (blue) cows; Table S1: Bacterial alpha-diversity analysis (genus level) of the microbial diversity in faeces from CON and TRT cows; Table S2: Bacterial beta-diversity analysis of faeces from treated and control cows over the eight sampling time points; Table S3: Linear discriminant analysis effects size (LEfSe) analysis (genus level) of faeces from CON compared to TRT cows over time; Table S4: Fungal Alpha-diversity analysis (genus level) of the microbial diversity in faeces from CON and TRT cows; Table S5: Fungal Beta-diversity analysis (genus level) of CON compared with TRT cows at individual time points within the study.

## References

[1] Henderson G., Cox F., Ganesh S., Jonker A., Young W., Janssen P.H. (2015). Rumen microbial community composition varies with diet and host, but a core microbiome is found across a wide geographical range. Sci. Rep..

[2] Kumar S., Indugu N., Vecchiarelli B., Pitta D.W. (2015). Associative patterns among anaerobic fungi, methanogenic archaea, and bacterial communities in response to changes in diet and age in the rumen of dairy cows. Front. Microbiol..

[3] Samtiya M., Aluko R.E., Dhewa T. (2020). Plant food anti-nutritional factors and their reduction strategies: An overview. Food Prod. Process. Nutr..

[4] Bickhart D., Weimer P. (2018). Symposium review: Host–rumen microbe interactions may be leveraged to improve the productivity of dairy cows. J. Dairy Sci..

[5] Zeineldin M., Barakat R., Elolimy A., Salem A.Z., Elghandour M.M., Monroy J.C. (2018). Synergetic action between the rumen microbiota and bovine health. Microb. Pathog..

[6] Janssen P.H., Kirs M. (2008). Structure of the archaeal community of the rumen. Appl. Environ. Microbiol..

[7] Stewart C., Flint H., Byrant M., Hobson P.N., Stewart C.S. (1997). The rumen bacteria. The Rumen Microbial Ecosystem.

[8] Dill-McFarland K.A., Weimer P.J., Pauli J.N., Peery M.Z., Suen G. (2016). Diet specialization selects for an unusual and simplified gut microbiota in two-and three-toed sloths. Environ. Microb..

[9] Deusch S., Camarinha-Silva A., Conrad J., Beifuss U., Rodehutscord M., Seifert J. (2017). A structural and functional elucidation of the rumen microbiome influenced by various diets and microenvironments. Front. Microbiol..

[10] Rinninella E., Raoul P., Cintoni M., Franceschi F., Miggiano G.A.D., Gasbarrini A., Mele M.C. (2019). What is the healthy gut microbiota composition? A changing ecosystem across age, environment, diet, and diseases. Microorganisms.

[11] Jami E., Israel A., Kotser A., Mizrahi I. (2013). Exploring the bovine rumen bacterial community from birth to adulthood. ISME J..

[12] Pitta D., Pinchak W., Dowd S., Dorton K., Yoon I., Min B., Fulford J., Wickersham T., Malinowski D. (2014). Longitudinal shifts in bacterial diversity and fermentation pattern in the rumen of steers grazing wheat pasture. Anaerobe.

[13] O′Hara E., Neves A.L., Song Y., Guan L.L. (2020). The role of the gut microbiome in cattle production and health: Driver or passenger?. Annu. Rev. Anim. Biosci..

[14] Krehbiel C.R., Rust S.R., Zhang G., Gilliland S.E. (2003). Bacterial direct-fed microbials in ruminant diets: Performance response and mode of action. J. Anim. Sci..

[15] Plaizier J., Mesgaran M.D., Derakhshani H., Golder H., Khafipour E., Kleen J., Lean I., Loor J., Penner G., Zebeli Q. (2018). Enhancing gastrointestinal health in dairy cows. Animal.

[16] Maslen B.N., Gray L.A., Ghorashi S.A., White J.D., Campbell M.A., Pant S.D. (2022). Temporal changes in the faecal microbiota of beef cattle on feedlot placement. Animals.

[17] Shanks O.C., Kelty C.A., Archibeque S., Jenkins M., Newton R.J., McLellan S.L., Huse S.M., Sogin M.L. (2011). Community structures of fecal bacteria in cattle from different animal feeding operations. Appl. Environ. Microbiol..

[18] Durso L., Wells J., Harhay G., Rice W., Kuehn L., Bono J., Shackelford S., Wheeler T., Smith T. (2012). Comparison of bacterial communities in faeces of beef cattle fed diets containing corn and wet distillers’ grain with solubles. Lett. Appl. Microbiol..

[19] Mccann J.C., Wickersham T.A., Loor J.J. (2014). High-throughput methods redefine the rumen microbiome and its relationship with nutrition and metabolism. Bioinform. Biol. Insights.

[20] Alawneh J.I., Barreto M.O., Moore R.J., Soust M., Al-Harbi H., James A.S., Krishnan D., Olchowy T.W. (2020). Systematic review of an intervention: The use of probiotics to improve health and productivity of calves. Prev. Vet. Med..

[21] Ramirez-Garzon O., Al-Alawneh J.I., Barber D., Liu H., Soust M. (2024). The effect of a direct fed microbial on liveweight and milk production in dairy cattle. Animals.

[22] Engelbrektson A., Kunin V., Wrighton K.C., Zvenigorodsky N., Chen F., Ochman H., Hugenholtz P. (2010). Experimental factors affecting PCR-based estimates of microbial species richness and evenness. ISME J..

[23] Alawneh J.I., Ramay H., Olchowy T., Allavena R., Soust M., Jassim R.A. (2024). Effect of a *Lactobacilli*-Based Direct-Fed Microbial Product on Gut Microbiota and Gastrointestinal Morphological Changes. Animals.

[24] Callahan B.J., McMurdie P.J., Rosen M.J., Han A.W., Johnson A.J.A., Holmes S.P. (2016). DADA2: High-resolution sample inference from Illumina amplicon data. Nat. Methods.

[25] Bolyen E., Rideout J.R., Dillon M.R., Bokulich N.A., Abnet C.C., Al-Ghalith G.A., Alexander H., Alm E.J., Arumugam M., Asnicar F. (2019). Reproducible, interactive, scalable and extensible microbiome data science using QIIME 2. Nat. Biotechnol..

[26] McDonald D., Jiang Y., Balaban M., Cantrell K., Zhu Q., Gonzalez A., Morton J.T., Nicolaou G., Parks D.H., Karst S.M. (2023). Greengenes2 unifies microbial data in a single reference tree. Nat. Biotechnol..

[27] Nilsson R.H., Larsson K.-H., Taylor A.F.S., Bengtsson-Palme J., Jeppesen T.S., Schigel D., Kennedy P., Picard K., Glöckner F.O., Tedersoo L. (2019). The UNITE database for molecular identification of fungi: Handling dark taxa and parallel taxonomic classifications. Nucleic Acids Res..

[28] Abarenkov K., Nilsson R.H., Larsson K.-H., Taylor A.F., May T.W., Frøslev T.G., Pawlowska J., Lindahl B., Põldmaa K., Truong C. (2024). The UNITE database for molecular identification and taxonomic communication of fungi and other eukaryotes: Sequences, taxa and classifications reconsidered. Nucleic Acids Res..

[29] Chao A. (1984). Nonparametric estimation of the number of classes in a population. Scand. J. Stat..

[30] Simpson E.H. (1949). Measurement of diversity. Nature.

[31] Hockett C.F., Shannon C.L., Weaver W. (1949). The Mathematical Theory of Communication.

[32] Jolliffe I.T., Cadima J. (2016). Principal component analysis: A review and recent developments. Philos. Trans. R. Soc. A Math. Phys. Eng. Sci..

[33] Rabinowitz G.B. (1975). An Introduction to Nonmetric Multidimensional Scaling. Am. J. Pol. Sci..

[34] Wickham H. (2016). Ggplot2: Elegant Graphics for Data Analysis.

[35] Posit Team (2023). RStudio: Integrated Development Environment for R. Posit Software.

[36] Oyebade A.O., Lee S., Sultana H., Arriola K., Duvalsaint E., Nino De Guzman C., Fernandez Marenchino I., Marroquin Pacheco L., Amaro F., Ghedin Ghizzi L. (2023). Effects of direct-fed microbial supplementation on performance and immune parameters of lactating dairy cows. J. Dairy Sci..

[37] Pupo M., Diepersloot E., Heinzen C., Ferraretto L. (2024). Dietary fiber source and direct-fed microbial supplementation effects on lactation performance and feeding behavior of high-producing dairy cows. J. Dairy Sci..

[38] Li J., Zhan S., Liu X., Lin Q., Jiang J., Li X. (2018). Divergence of Fecal Microbiota and Their Associations with Host Phylogeny in Cervinae. Front. Microbiol..

[39] Magne F., Gotteland M., Gauthier L., Zazueta A., Pesoa S., Navarrete P., Balamurugan R. (2020). The Firmicutes/Bacteroidetes ratio: A relevant marker of gut dysbiosis in obese patients?. Nutrients.

[40] Fernandes K.A., Rogers C.W., Gee E.K., Kittelmann S., Bolwell C.F., Bermingham E.N., Biggs P.J., Thomas D.G. (2021). Resilience of faecal microbiota in stabled thoroughbred horses following abrupt dietary transition between freshly cut pasture and three forage-based diets. Animals.

[41] Bartenslager A. (2020). Investigating Microbiomes and Developing Direct-fed Microbials to Improve Cattle Health. Master’s Thesis.

[42] Dankwa A., Humagain U., Ishaq S., Yeoman C., Clark S., Beitz D., Testroet E. (2021). Bacterial communities in the rumen and feces of lactating Holstein dairy cows are not affected when fed reduced-fat dried distillers’ grains with solubles. Animal.

[43] Wang X., Tsai T., Wei X., Zuo B., Davis E., Rehberger T., Hernandez S., Jochems E.J., Maxwell C.V., Zhao J. (2021). Effect of Lactylate and *Bacillus subtilis* on growth performance, peripheral blood cell profile, and gut microbiota of nursery pigs. Microorganisms.

[44] Varga G.A., Kolver E.S. (1997). Microbial and animal limitations to fiber digestion and utilization. J. Nutr..

[45] De Vos C. (2019). Effect of Feed Additive Supplementation on Rumen Bacterial Amino Acid Profile and Fermentation Dynamics in Dairy Cows. Master’s Thesis.

[46] Ban Y., Guan L.L. (2021). Implication and challenges of direct-fed microbial supplementation to improve ruminant production and health. J. Anim. Sci. Biotechnol..

[47] Russell J.B., Rychlik J.L. (2001). Factors that alter rumen microbial ecology. Science.

[48] Li S., Young T., Archer S., Lee K., Sharma S., Alfaro A.C. (2022). Mapping the green-lipped mussel (*Perna canaliculus*) microbiome: A multi-tissue analysis of bacterial and fungal diversity. Curr. Microbiol..

[49] Yin X.-J., Ji S.-K., Duan C.-H., Tian P.-Z., Ju S.-S., Yan H., Zhang Y.-J., Liu Y.-Q. (2022). Dynamic change of fungal community in the gastrointestinal tract of growing lambs. J. Integr. Agric..

[50] Tian Q., Chomnunti P., Lumyong S., Liu J., Hyde K. (2021). Phylogenetic relationships and morphological reappraisal of Chaetothyriales. Mycosphere.

[51] Svantesson S., Kõljalg U., Wurzbacher C., Saar I., Larsson K.-H., Larsson E. (2021). *Polyozellus* vs. *Pseudotomentella*: Generic delimitation with a multi-gene dataset. Fungal Syst. Evol..

[52] Imdad S., So B., Jang J., Park J., Lee S.-J., Kim J.-H., Kang C. (2024). Temporal variations in the gut microbial diversity in response to high-fat diet and exercise. Sci. Rep..

[53] Noel S.J., Olijhoek D.W., Mclean F., Løvendahl P., Lund P., Højberg O. (2019). Rumen and fecal microbial community structure of Holstein and Jersey dairy cows as affected by breed, diet, and residual feed intake. Animals.

[54] Kibegwa F.M., Bett R.C., Gachuiri C.K., Machuka E., Stomeo F., Mujibi F.D. (2023). Diversity and functional analysis of rumen and fecal microbial communities associated with dietary changes in crossbreed dairy cattle. PLoS ONE.

[55] Nocek J.E., Kautz W.P., Leedle J.A.Z., Allman J.G. (2002). Ruminal Supplementation of Direct-Fed Microbials on Diurnal pH Variation and In Situ Digestion in Dairy Cattle. J. Dairy Sci..

