# Supplementary material for: Temporal Changes in Faecal Microbiota Composition and Diversity in Dairy Cows Supplemented with a Lactobacillus-Based Direct-Fed Microbial
Source: Animals (Basel). 2024 Nov 27;14(23):3437. doi: 10.3390/ani14233437 (PMC11640597; doi:10.3390/ani14233437)
Supplement: Supplementary file 1 [file animals-14-03437-s001.zip › animals-3267556-supplementary.pdf]

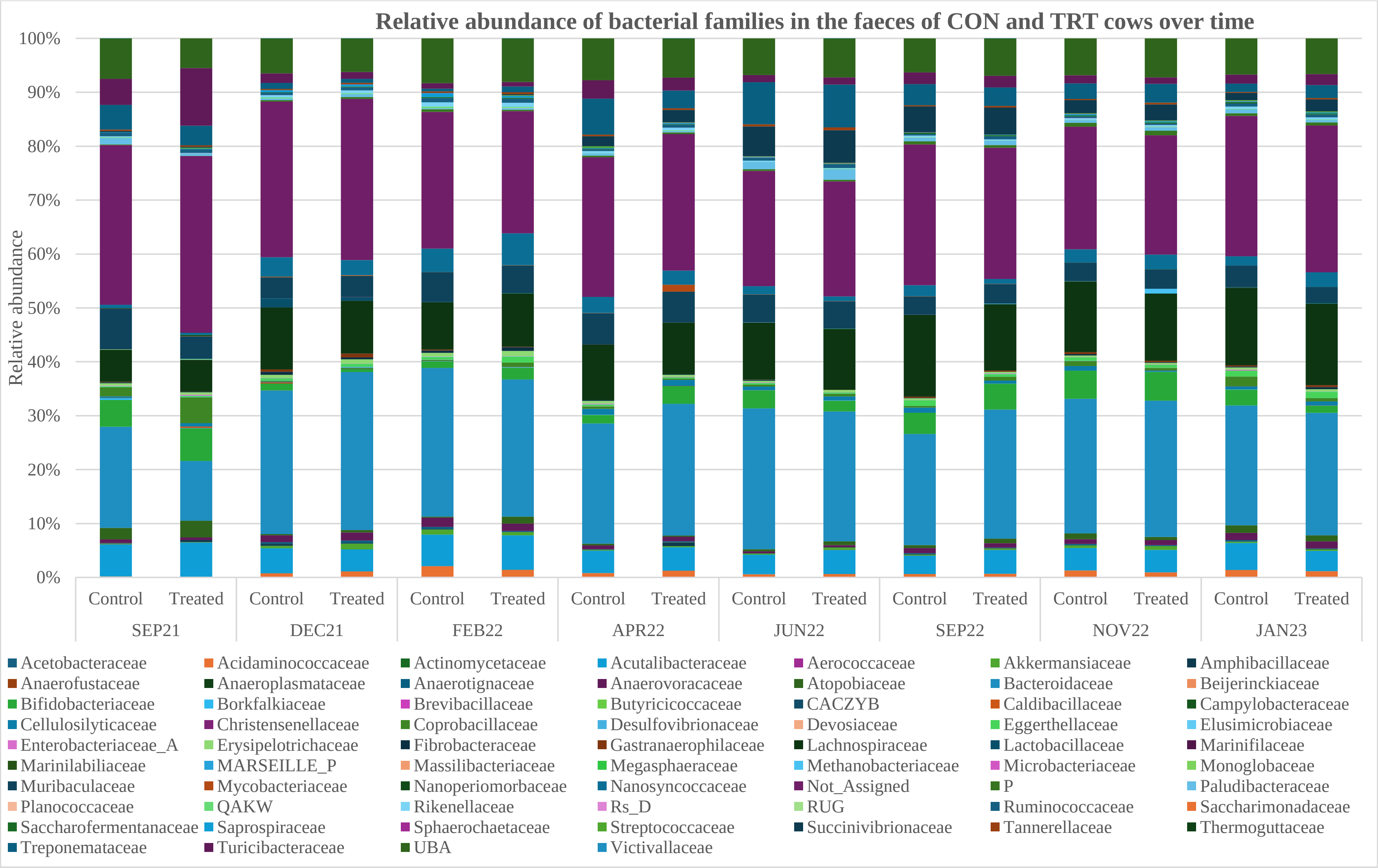

Figure S1: Relative abundance of bacterial families in faeces for CON and TRT groups throughout the study period.

Table S1: Bacterial alpha-diversity analysis (genus level) of the microbial diversity in faeces from CON and TRT cows. Statistically significant p-values, indicating difference between CON and TRT cow microbiotas, are highlighted in blue and asterisked.

Months	Observed		Chao1		Shannon	
	**p-value**	**T-test**	**p-value**	**T-test**	**p-value**	**T-test**
September 2021	0.002*	3.3	0.002*	3.3	0.16	-1.4
December 2021	0.19	-1.3	0.20	-1.3	0.02*	-2.4
February 2022	0.51	-0.7	0.51	-0.7	0.38	-0.9
April 2022	0.31	1.0	0.31	1.0	0.08	1.8
June 2022	0.57	0.6	0.58	0.6	0.80	0.3
September 2022	0.73	0.3	0.73	0.3	0.76	-0.3
November 2022	0.74	0.3	0.74	0.3	0.26	-1.2
January 2023	0.07	-1.9	0.07	-1.9	0.002*	-3.3

* a probability value ≤ 0.05 is deemed significant

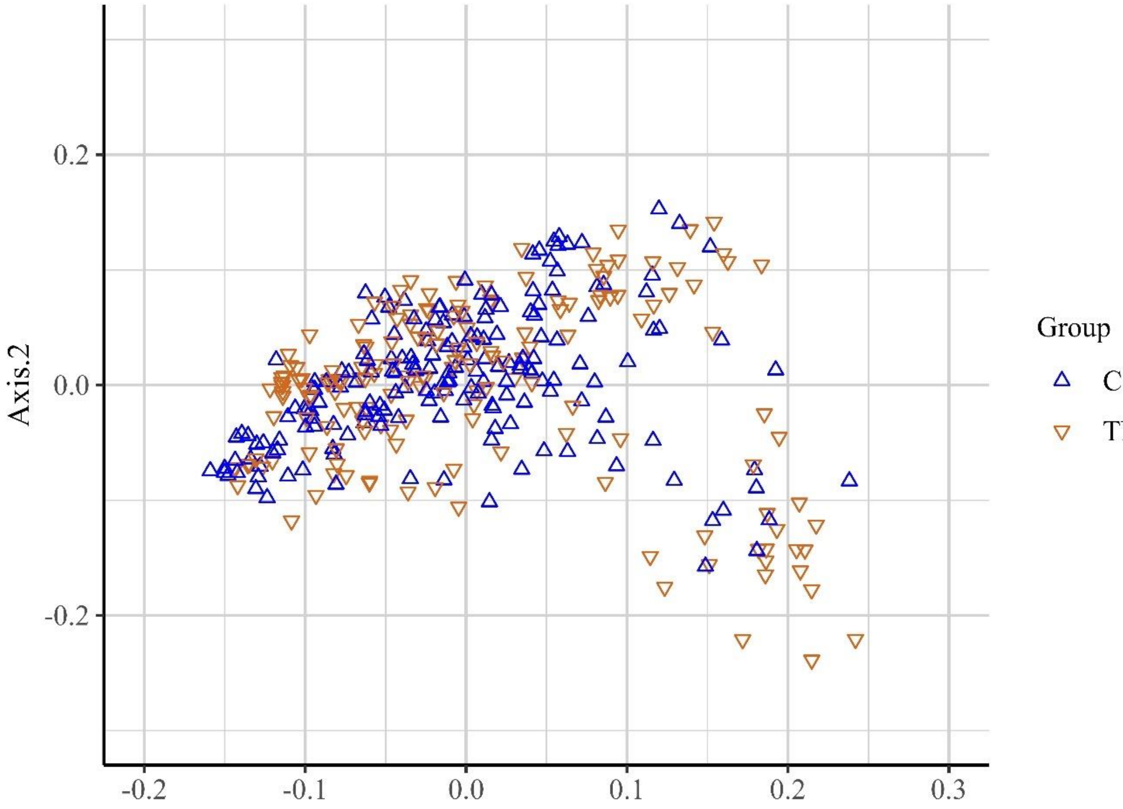

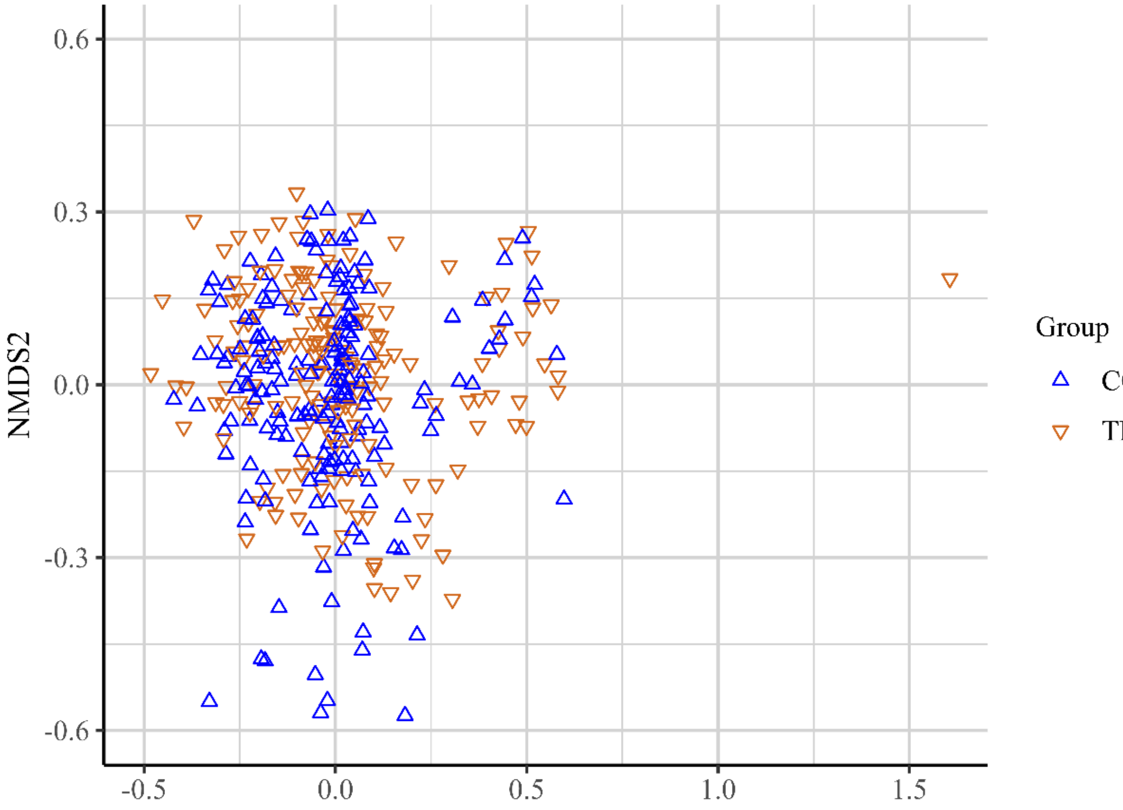

Figure S2: Bacterial beta-diversity (genus level) analysis of faeces from TRT and CON cows. Treatment had significant effects on the microbial diversity of faeces. F-value: 3.0, R^2: 0.008, p-value: 0.007, Stress: 0.23.

Table S2: Bacterial beta-diversity analysis of faeces from treated and control cows over the eight sampling time points.

Months	F-value	R^2	p-value	Stress
September 2021	7.4	0.14	0.001*	0.16
December 2021	6.1	0.12	0.001*	0.25
February 2022	4.2	0.09	0.001*	0.24
April 2022	3.2	0.07	0.001*	0.24
June 2022	2.0	0.05	0.018*	0.24
September 2022	6.2	0.13	0.001*	0.26
November 2022	2.7	0.06	0.002*	0.26
January 2023	4.5	0.10	0.001*	0.27

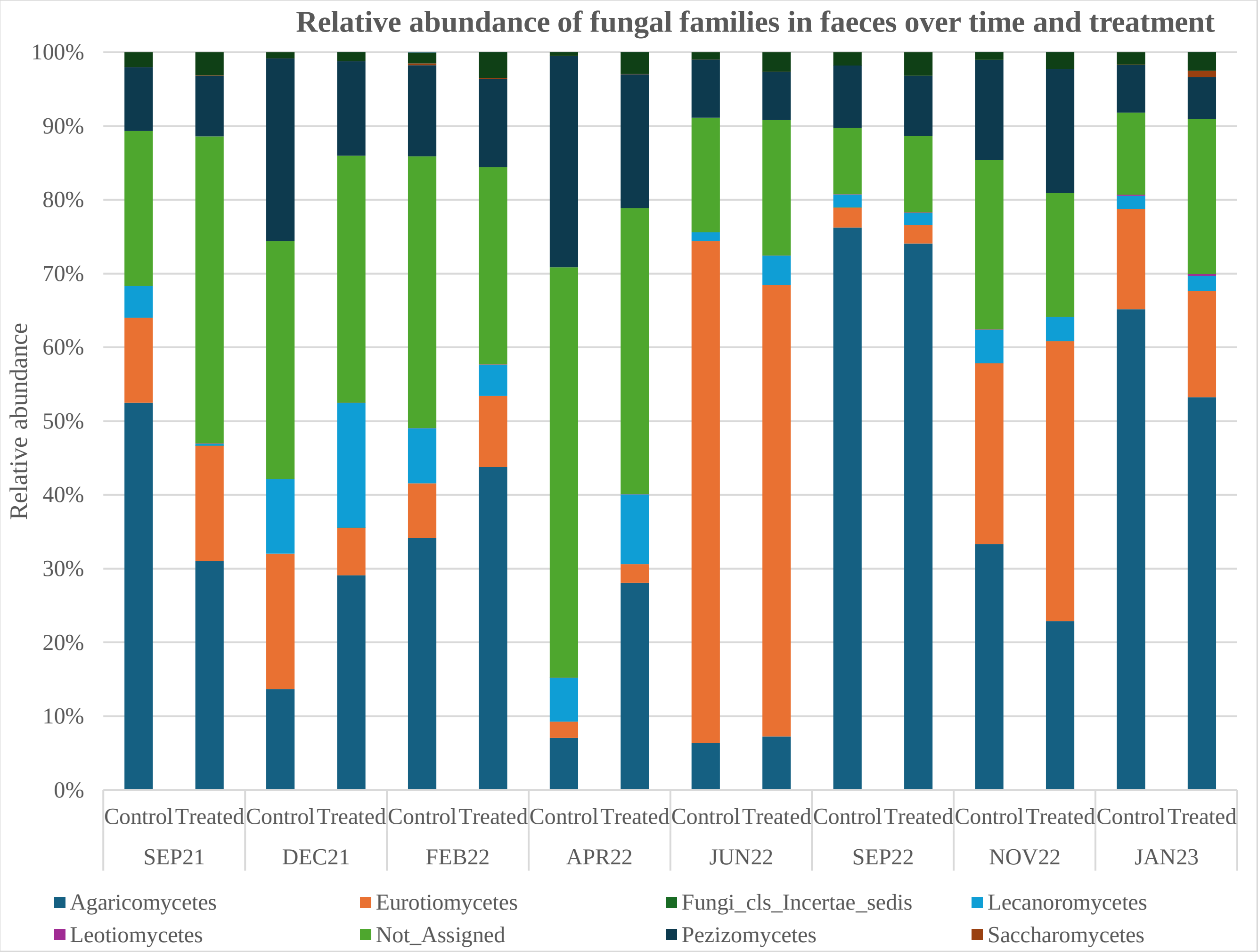

Figure S3: Relative abundance of fungal classes in faeces for CON and treatment TRT groups throughout the study period.

Table S3: Linear discriminant analysis effects size (LEfSe) analysis (genus level) of faeces from CON compared to TRT cows over time.

Genus	P-value	FDR	Control	Treated	LDAscore
**September 2021**					
Paraprevotella	1.44E-06	0.0002	594410	232180	5.3
Ruminococcus_C_58660	5.37E-05	0.003	6859	41437	-4.2
Caldibacillus_295372	8.34E-05	0.003	5600	28795	-4.1
Turicibacter	8.44E-05	0.003	520170	1113200	-5.5
Bacillus_P_294101	9.41E-05	0.003	404	9255	-3.7
Clostridium_T	0.0002	0.004	309290	644670	-5.2
Virgibacillus_E_287324	0.0002	0.005	4067	16063	-3.8
Oceanobacillus	0.0003	0.006	6538	23365	-3.9
CAG_485	0.0007	0.01	126550	69851	4.5
Paramuribaculum	0.001	0.02	379570	174700	5.0
UBA4334	0.001	0.02	73796	22259	4.4
Sharpea	0.002	0.02	166180	449310	-5.2
Cellulosilyticum	0.002	0.02	27377	61240	-4.2
CCUG_7971	0.002	0.02	7891	15759	-3.6
Phascolarctobacterium_A	0.002	0.02	18100	4110	3.8
UBA4285	0.002	0.02	1058	9785	-3.6
Alloprevotella	0.003	0.03	122980	40409	4.6
RF16	0.004	0.03	107740	32403	4.6
UBA7741	0.005	0.04	36408	78351	-4.3
Phocaeicola_A_858004	0.006	0.04	614450	400590	5.0
Eubacterium_O_258268	0.007	0.05	0	2108	-3.0
**December 2021**					
Bacillus_P_294101	2.62E-09	3.96E-07	72212	15843	4.5
Ornithinibacillus_287146	3.76E-06	0.0003	5123.8	828	3.3
Akkermansia	1.08E-05	0.0005	43274	101950	-4.5
Oceanobacillus	0.0003	0.01	31466	14316	3.9
Phocaeicola_A_858004	0.0005	0.01	915160	1081300	-4.9
Clostridium_T	0.0005	0.01	102130	59030	4.3
Mailhella	0.0005	0.01	2761	18769	-3.9
Ruminococcus_E	0.0007	0.01	167340	77797	4.7
Limosilactobacillus	0.001	0.02	183240	78380	4.7
CAG_791	0.001	0.02	506910	347360	4.9
**February 2022**					
Fructilactobacillus	1.79E-05	0.003	5641	0	3.5
RUG420	4.44E-05	0.003	78720	34624	4.3
OLB9	6.76E-05	0.003	74668	30427	4.3
BICA1_8	0.0002	0.006	32878	16741	3.9
Anaerotignum_189125	0.0006	0.02	47017	20396	4.1
Parafannyhessea	0.0007	0.02	0	82807	-4.6
UBA737	0.0007	0.02	121040	247520	-4.8

Phascolarctobacterium_A	0.001	0.02	246110	148150	4.7
UBA2658	0.001	0.02	15207	1622	3.8
Not_Assigned	0.003	0.04	3734400	3409600	5.2
CAG_83	0.003	0.04	41989	21809	4.0
UBA11471	0.003	0.04	19598	8900	3.7
**April 2022**					
Oceanobacillus	1.31E-09	1.67E-07	62	71758	-4.6
Corynebacterium	1.30E-08	8.24E-07	968	146140	-4.9
Ornithinibacillus_287146	9.28E-05	0.004	0	3039	-3.2
**June 2022**					
NOSIGNIFICANTFEATURES					
**September 2022**					
Prevotella	2.83E-08	4.75E-06	48428	163180	-4.8
Sharpea	2.93E-06	0.0002	17860	65772	-4.4
UBA2834	9.35E-06	0.0005	58042	18751	4.3
UBA9715	5.30E-05	0.002	82622	36671	4.4
Nanosyncoccus	6.52E-05	0.002	147510	73795	4.6
Faecousia	0.0001	0.004	720830	832010	-4.8
Choladousia	0.0003	0.007	6076	15614	-3.7
UBA737	0.0009	0.02	106700	184790	-4.6
Agathobacter_164119	0.001	0.02	401780	225930	4.9
QALR01	0.002	0.03	0	2009	-3.0
UBA1394	0.002	0.03	0	3629	-3.3
Faecalimonas	0.002	0.03	30325	56918	-4.1
Parafannyhessea	0.003	0.03	46706	76685	-4.2
Eubacterium	0.004	0.04	20132	7652	3.8
Phocaeicola_A_858004	0.004	0.04	786200	896270	-4.7
Parabacteroides_B_862066	0.004	0.04	20184	33390	-3.8
CAG_269	0.005	0.04	18777	35053	-3.9
Not_Assigned	0.005	0.05	2830700	2531800	5.2
RUG420	0.006	0.05	42823	31525	3.8
**November 2022**					
Cellulosilyticum	5.56E-05	0.008	80133	16206	4.5
Berryella	0.0007	0.05	8397	0	3.6
UMGS1071	0.001	0.05	1154	7619	-3.5
**January 2023**					
Paramuribaculum	2.89E-05	0.003	207410	123960	4.6
Berryella	4.46E-05	0.003	19664	1749	4.0
Fructilactobacillus	6.18E-05	0.003	9879.9	0	3.7
UBA737	0.0003	0.01	141310	82147	4.5
Faecalimonas	0.0007	0.02	79327	40863	4.3
Sharpea	0.001	0.03	157850	49719	4.7
BICA1_8	0.002	0.03	2733	8390	-3.5
Nanosyncoccus	0.002	0.03	172610	259280	-4.6
Hornefia	0.002	0.03	7292	19136	-3.8
Bifidobacterium_388775	0.002	0.03	323580	146570	5.0

UMGS1071	0.003	0.04	347	4594	-3.3

P-value is the probability value, FDR is the false discovery rate and LDA score is the linear discriminant analysis score.

Table S4: Fungal Alpha-diversity analysis (genus level) of the microbial diversity in faeces from CON and TRT cows.

	**Observed**		**Chao1**		**Shannon**	
	**p-value**	**T-test**	**p-value**	**T-test**	**p-value**	**T-test**
September 2021	0.79	-0.27	0.79	-0.27	0.04*	2.2
December 2021	0.53	0.64	0.53	0.64	0.0003*	3.9
February 2022	0.32	1.00	0.32	1.00	0.01*	2.8
April 2022	0.37	0.91	0.37	0.91	0.01*	3.6
June 2022	0.32	-1.01	0.32	-1.01	0.02*	2.5
September 2022	0.38	-0.88	0.38	-0.88	0.86	0.2
November 2022	0.09	-1.76	0.09	-1.76	3.38E-06*	-5.5
January 2023	0.63	-0.48	0.63	-0.48	0.25	1.2

* a probability value ≤ 0.05 is deemed significant

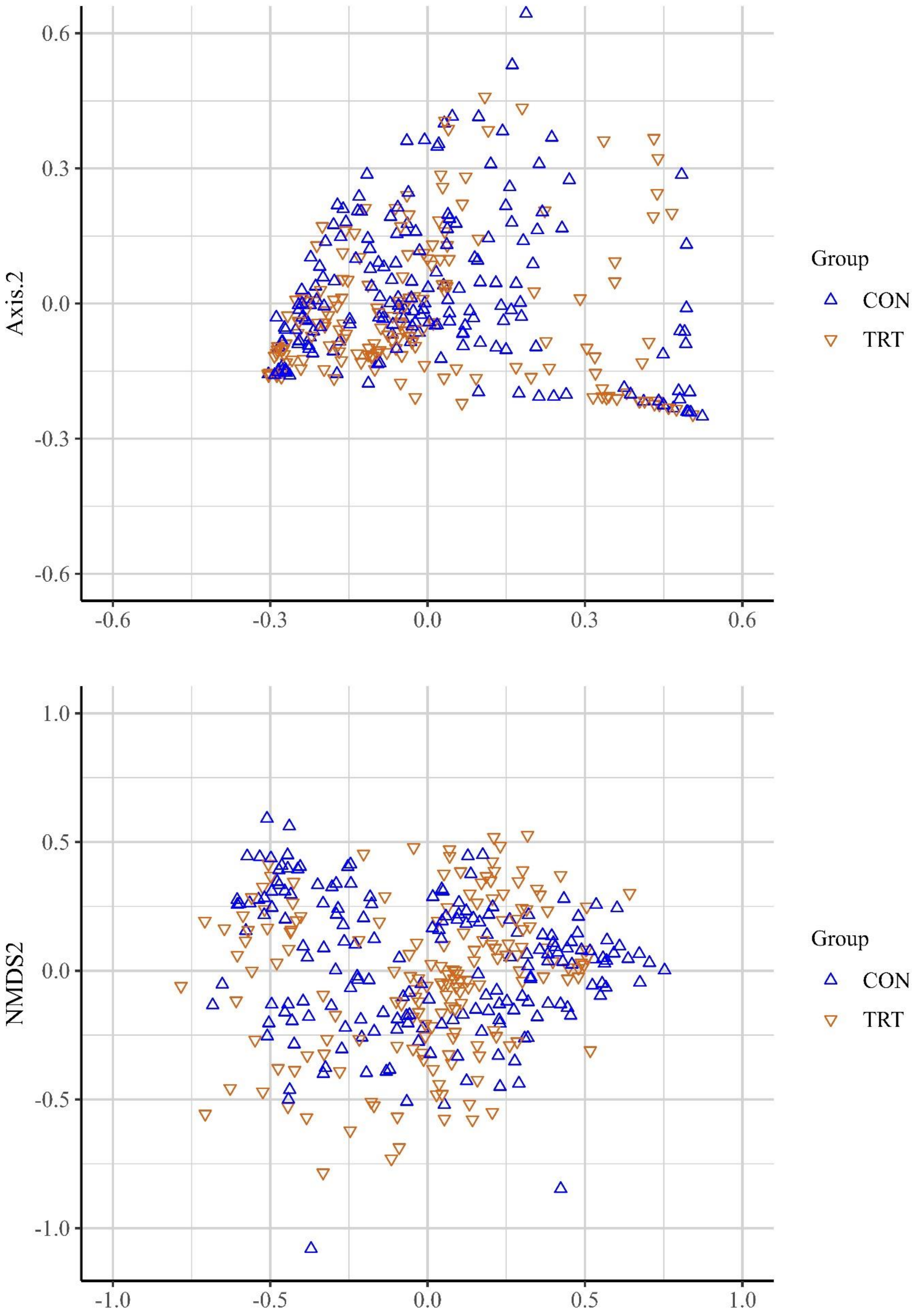

Figure S4: Fungal Beta-diversity analysis (genus-level) of fungal diversity between CON (orange) and TRT (blue) cows. The treatment regime with/without had no significant impact on fungal diversity in faeces. F-value: 2.3, R^2: 0.006, p-value: 0.067-0.071, Stress: 0.23.

Table S5: Fungal Beta-diversity analysis (genus level) of CON compared with TRT cows at individual time points within the study.

	**F-value**	R^2	**p-value**	**Stress**
September 2021	17.3	0.3	0.001*	0.14
December 2021	28.5	0.4	0.001*	0.16
February 2022	9.0	0.2	0.001*	0.20
April 2022	1.9	0.05	0.14	0.24
June 2022	3.2	0.1	0.048*	0.19
September 2022	4.8	0.1	0.002*	0.04
November 2022	9.0	0.2	0.001*	0.18
January 2023	14.4	0.3	0.001*	0.17

* a probability value ≤ 0.05 is deemed significant
